# Progress in Psychiatry

**Published:** 1901-06-29

**Authors:** 


### Progress in Psychiatry.

(*Continued from page* 197.)

**General Paralysis of the Insane.**—This is a disease which is one of the gravest and most dangerous forms of insanity, hopeless in prognosis, progressive in character, and almost invariably fatal within a few years of the onset of mental symptoms. It is also known under the names of progressive paralysis, or paralytic dementia. Pathologically it is an acute or subacute form of cerebral degeneration (meningo-encephalitis), diffuse and extensive in distribution, often associated with degenerations of special groups of bulbo-spinal neurous, owning an ætiology of a parasyphilitic or alcoholic nature operating on a hereditary basis of a gross neurotic, neuro-vascular, or vesanic character. Its onset is subtle and insidious; it developes on a basis of combined cerebral toxæmia, sexual and alcoholic excess, and mostly frequently in persons of fine physical development, of ardent temperament, and of strong animal and sexual appetites. The type of individual who is by nature inclined to the ascetic and quieter forms of living seldom develops general paralysis of the insane, although he may become the subject of other forms of mental disease. Tendencies to chronic renal disease and arterio-sclerosis are often present in the subjects of general paralysis or in their parents and collaterals Hence also cerebral hæmorrhage, thrombosis, and cerebral paralysis are often met with among the immediate ancestors of general paralytics. The form of heredity which here is in question is that designated by French observers a "cerebral-congestive" or "neuro-vascular" heredity. A vesanic heredity, apart from the cerebral kind just mentioned, is now accepted as proved to occur in a certain proportion of cases of general paralysis (Wahl,[92] Bechet[93]). The ætiology of general paralysis is complex, and is regarded differently by various observers. Charcot speaks of a neurotic predisposition to general paralysis, probably inherited, and regards syphilis as an exciting cause. Other causes, such as alcohol and cerebral over-exertion, are named by him as factors capable of exciting general paralysis without the co-operation of syphilis.

Quite recently Marandon de Montyel[94] has brought evidence to show that paludism may be in certain cases a cause of general paralysis. Whatever causes are capable of creating prolonged states of cerebral hyperæmia may lead to diffuse meningo-encephalitis, especially if renal disease is at the same time present. In this connection it is interesting to note that a certain toxicity of the blood will predispose to the apoplectiform seizures of general paralysis (Magnan) as it does to attacks of status epilepticus (Herter, Cololian[95]) or of acute mania (D'Abundo). This toxæmia may arise from various causes most of which are at present obscure. The action of alcohol and alcoholic liquors, of absinthe, of lead, of parasyphilitic toxins, of toxic agencies of malarial origin, and of deleterious substances accumulated in the system during renal disease, may severally or conjointly act in bringing about manifestations of general paralysis. Acquired syphilis seems to be by far the commonest antecedent of general paralysis occurring in the adult. Moreover, nearly every case of juvenile general paralysis (Alzheimer,[96] Thiry[97]) known has been found to be the subject of congenital syphilis. The figures given by the two last-mentioned authors are as follows:—Out of 35 cases of juvenile general paralysis in which the symptoms were carefully investigated there was syphilis in 27 (23 being congenital and 4 acquired), and in the remaining 8 it was at least probable (Alzheimer). The statistics of Thiry show that in 90 per cent. of cases of juvenvile general paralysis there could be traced a history of congenital syphilis.

At the Charite Hospital, Berlin, the records of female general paralytic patients admitted during the years 1881–1892 show that 51 per cent. of them had had syphilis. Rieger, of Wurzburg, from a study of 1,000 cases of insanity in its various forms, found that whereas only 40 non-paralytic patients had a history of syphilis, 400 general paralytics had a similar history; or that acquired syphilis as a condition preceding the insanity was ten times more frequent in general paralysis than in the other insanities. General paralysis as a form of insanity is common among military officers and rare among ecclesiastics. Thus Kundt stated that among 1,090 insane patients there were 16 Catholic priests, not one of whom was a general paralytic, and 13 military officers, 8 of whom were general paralytics. The correlation or frequent association of general paralysis with the prevalence of syphilis, drink, and general fastness of living in the military profession, has been also pointed out by many authors, including Fournier, Krafft-Ebing, and Mickle. Krafft-Ebing, of Vienna, attempted to inoculate 8 cases of general paralysis with secretions taken from fresh syphilitic chancres, but without success, so that he concluded that these patients were already "syphilised," or had latent syphilis at the time of the experimental inoculation. This deduction, however, is not conclusive. Indeed, that author caustically observes that syphilisation and civilisation are the factors which co-operate to produce general paralysis. Cerebral traumatism is occasionally the exciting cause of general paralysis, and the same has been claimed for sunstroke, but here, too, the exact *modus operandi* has not been traced.[98] Joffroy has personally observed 10 cases of general paralysis, the parents of whom suffered from the same complaint; Oscar Müller, Wahl, Vallon, and Turnbull have also recorded such, cases. Scarlatina in childhood has been noted by Gianelli as a co-operating cause in the production of general paralysis, other conditions being favourable. Thus in the case of a girl aged 7 years with a history of syphilitic and alcoholic parentage, an attack of scarlatina with nephritis was rapidly followed by signs of commencing dementia. Her disposition changed, she developed a fine general tremor, and later had epileptiform attacks and dementia, in which condition she died. An autopsy, though an incomplete one, was made and it verified the diagnosis of general paralysis.

So far as our knowledge of geographical pathology goes savage nations and tribes have not as yet attained to the level of development which permits of the manifestation of general paralysis. The Arabs, the native Egyptians, and other primitive races were till quite recently free of this disease, though syphilis is freely prevalent both among the savage races of Africa and the Arabs proper. The Celt in his primitive home (Ireland, Wales, the Highlands) is free of general paralysis, but with civilisation he acquires a proclivity to this disease. The negro in the United States similarly seems to have acquired civilisation and general paralysis *pari passu*. In agricultural country districts and villages, with their quiet unexciting life, general paralysis seldom makes its appearance, but in the great seaport towns and in mining and manufacturing centres where alcohol, syphilis, and the excitements and stresses of life are at their height, general paralysis is rife. Under the latter conditions of life no race seems to be exempt: the Celt as well as the Saxon, the Slav as well as the Gaul, and even the American negro are among the victims. The American negro, says Berkley, affords a striking example of the acquisition of general paralysis with "civilisation and syphilisation." Before the civil war in the United States, and for some few years afterwards, the disease was unknown among the American negroes. Little by little the number of cases grew in frequency. Such patients were at first regarded as curiosities, "but at present (1900) in Baltimore paretics represent approximately the same percentage, according to the general population, in negroes as they do among the whites; nor do the general types of the disease differ materially in the two races." The increase of general paralysis in the female population of our large manufacturing and mining centres and seaport towns is significant and ominous. While exceedingly rare among gentlewomen (Mickle, Clouston), it has increased among the lower and working classes of females, and this applies to both married and single women. The diminishing ratio of general paralysis in women of the private class, as shown from the returns of private asylums, is due, according to Stewart,[99] to the absence of alcoholic excess. The increase of general paralysis is according to the reports of the Commissioners in Lunacy for the last few years most pronounced in large towns, and is greatest of all in seaport towns, and next greatest in mining counties (Newcastle, Cardiff, Durham, Yorkshire, Lancashire). In agricultural counties generally there is an actual diminution.

The average annual number of general paralytics admitted to English asylums during the five years 1894–98 was 1,400, or 7·6 per cent. of all the insane. Ireland, Scotland, Sweden, Italy, Spain, Turkey, and Greece enjoy a relative immunity from general paralysis, whereas it thrives and flourishes in England, France, Germany, and the United States. Among famous or distinguished persons who have died of generel paralysis must be included Sir Edwin Landseer the painter, Ascher the musician, Guy de Maupassant the novelist, Lord Randolph Churchill, and the painter Munkacszy.

[92] Thèse de Paris, 1898, and Brit. Med. Jour., Epit. No. 209, Mar. 25, 1899. [93] Thèse de Paris, 1897. [94] Paludism as a cause of general paralysis, Archives de Neurologie, 1901. [95] Archives de Neurolagie, Mar. 1899. [96] Neurolog. Central blatt, Oct. 15, 1894. [97] Thèse de Lyon, 1896. [98] Meschede, in Allgem. Leitschr. für Psychiatic, Feb. 1899. [99] Jour. of Ment. Sci., 1895.

